# Flache pigmentierte Läsionen in UV-exponierter Haut – eine diagnostische Herausforderung

**DOI:** 10.1007/s00105-021-04846-w

**Published:** 2021-06-26

**Authors:** Teresa Deinlein, Rainer Hofmann-Wellenhof, Andreas Blum

**Affiliations:** 1 Universitätsklinik für Dermatologie und Venerologie, Medizinische Universität Graz, Auenbruggerplatz 8, 8036 Graz, Österreich; 2 Hautarzt- und Lehrpraxis, Konstanz, Deutschland

Im klinischen Alltag stellen flache pigmentierte Läsionen in UV-exponierter Haut nicht selten klinisch und dermatoskopisch eine Herausforderung dar, insbesondere wenn sie solitär stehen oder die exakte Anamnese nicht sicher zu erheben ist. Diese Problematik soll anhand von 4 Läsionen im Folgenden gezeigt und diskutiert werden.

## Fall 1

**Figure 1:**
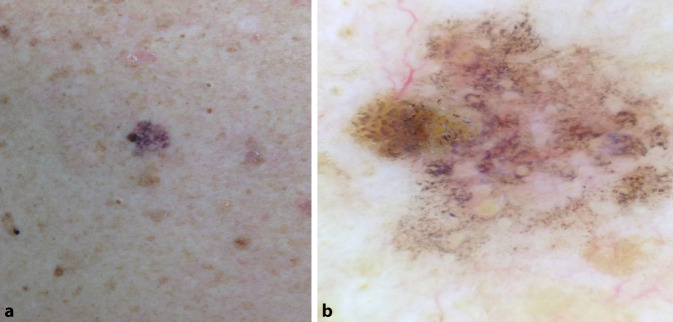

Ein 62 Jahre alter Patient, an den Schultern Veränderung von Form und Farbe bemerkt (Abb. 1).

## Fall 2

**Figure 2:**
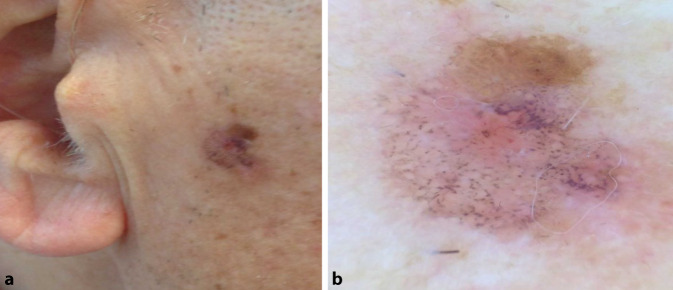

Ein 68 Jahre alter Patient, an der rechten Wange Veränderung von Form und Farbe bemerkt (Abb. 2).

## Fall 3

**Figure 3:**
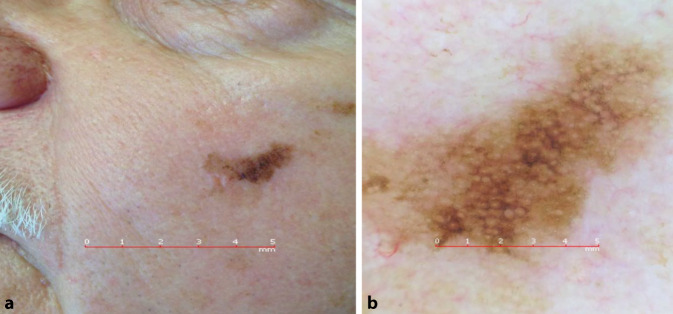

Ein 74 Jahre alter Patient, an der linken Wange, keine Veränderung bemerkt (Abb. 3).

## Fall 4

**Figure 4:**
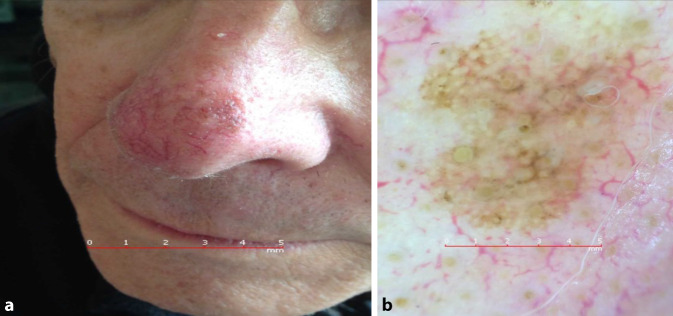

Ein 78 Jahre alter Patient, an der linken Nasenspitze, keine Veränderung bemerkt (Abb. 4).

## Wie lautet Ihre Diagnose?

**Diagnose:** Fall 1: Lichen-planus-artige Keratose

**Figure 5:**
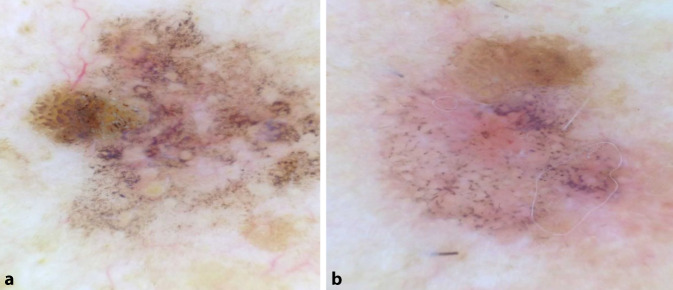

Dermatoskopisch zeigt sich eine relativ scharf begrenzte, bräunlich-livide, teilweise rötliche Makula mit einem exzentrisch gelegenen braunen Anteil bei 3 Uhr (Abb. 5a). Auffallend ist bei diesem Patienten außerdem die ausgeprägte aktinische Schädigung in der Umgebung der Läsion. In der Dermatoskopie sind unzählige grau-schwarze, über nahezu die gesamte Läsion verteilte Punkte erkennbar. Zudem findet man einzelne unregelmäßig pigmentierte Haarfollikel und am linken mittleren Rand ein hellbraunes Areal mit mehreren mittelbraunen gebogenen Linien.

**Diagnose:** Fall 2: Lichen-planus-artige Keratose

Unregelmäßig begrenzte, bräunlich-livide Makula mit einem mittelbraunen, exzentrischen Areal am apikalen Rand und einer kleinen Erosion im Zentrum (Abb. 5b). Dermatoskopisch finden sich auch hier unzählige grau-schwarze Punkte über nahezu die gesamte Läsion sowie ein homogen mittelbraunes Areal mit einzelnen gebogenen Linien im apikalen Anteil.

**Diagnose:** Fall 3: Lentigo maligna

**Figure 6:**
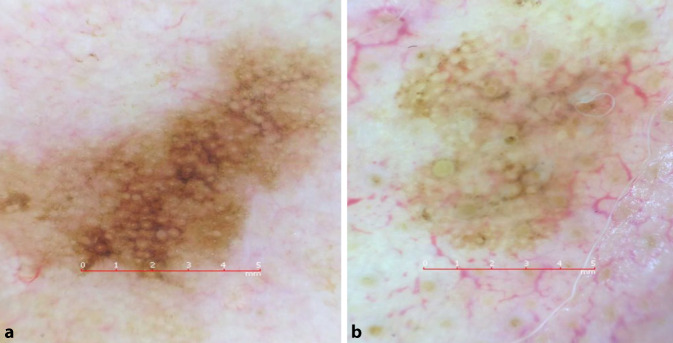

Unregelmäßig begrenzte, längliche Makula mit verschiedenen Brauntönen sowie kleinen schwarzen Arealen (Abb. 6a). In der Dermatoskopie sind multiple unregelmäßig pigmentierte Haarfollikel zu erkennen, die sich zum Teil als graue Kreise präsentieren. Manche dieser Kreise sind zweischichtig aufgebaut und ergeben das Bild eines „circle within a circle“.

**Diagnose:** Fall 4: Lentigo maligna

Klinisch unscheinbare hellbraune Makula an der linken Nasenspitze. Dermatoskopisch sind mehrere unregelmäßig pigmentierte Haarfollikel, gräuliche Punkte und auch zweischichtig aufgebaute Kreise zu erkennen (Abb. 6b).

## Diskussion

Die Lichen-planus-artige Keratose (LPLK), auch bekannt als lichenoide Keratose, ist eine häufig diagnostizierte gutartige Entität. Das Wissen um ihre klinischen und v. a. dermatoskopischen Muster ist von besonderer Relevanz, da sie aufgrund überlappender Kriterien eine häufige Differenzialdiagnose von malignen Melanomen darstellt [1–6].

Es wird angenommen, dass es sich bei der LPLK um einen dynamischen inflammatorischen Prozess handelt, an dessen Ende die vollständige Regression der vorbestehenden Läsion (meist eine solare Lentigo oder seborrhoische Keratose) steht [1]. Im letzten Stadium findet man dermatoskopisch ausschließlich gräuliche Punkte diffus über die Läsion verteilt, sodass eine Abgrenzung zu anderen vollständig regressiven Tumoren (z. B. dem malignen Melanom) nicht mehr sicher möglich ist [1].

Mehrere Studien haben sich mit den dermatoskopischen Mustern von LPLK und deren Abgrenzung v. a. zum malignen Melanom beschäftigt [1–5].

LPLK zeigen, abhängig vom Stadium der Regression, überwiegend ein hellbraunes Pseudonetzwerk, einen rosafarbenen Hintergrund, anuläre-granuläre Strukturen und ein graues Pseudonetzwerk (bestehend aus diffus verteilten blau-grauen Punkten) [1].

Eine große retrospektive Studie [2] an 473 histologisch untersuchten Läsionen (355 LPLK und 118 Nicht-LPLK) wies nach, dass LPLK signifikant häufiger eine Schuppung sowie orangefarbene Areale zeigten. Falls blau-graue Punkte („peppering“) als Zeichen der Regression vorhanden waren, so waren diese bei LPLK eher grobkörniger als bei Nicht-LPLK. Zudem konnten die Autoren zeigen, dass blau-graue Punkte als einziges dermatoskopisches Muster ein starker Hinweis für das Vorliegen einer LPLK sind und dass über die Hälfte der Läsionen Gefäße (vorwiegend Punktgefäße) zeigte. In der Subgruppenanalyse, in der die pigmentierten Läsionen gesondert ausgewertet wurden, wurden die oben genannten Kriterien ebenfalls signifikant häufiger bei LPLK gefunden.

In einer weiteren Arbeit [3] wurde gezeigt, dass ein Großteil der LPLK ein dermatoskopisches Muster zeigte (strukturlos, Punkte oder gewinkelte Linien). Waren mehrere Muster vorhanden, so fanden die Autoren vorwiegend die Kombinationen aus strukturlos + Punkte und strukturlos + gewinkelte Linien. Über alle Gruppen betrachtet, waren auch in dieser Studie graue Punkte das am häufigsten gefundene Kriterium.

Die Diagnose einer Lentigo maligna stellt weiterhin eine diagnostische Herausforderung dar, da die dermatoskopischen Kriterien gerade bei frühen Läsionen sehr subtil sein können [4]. Im Jahr 2000 beschrieb die Gruppe um Stolz [5] erstmals ein Progressionsmodell der Lentigo maligna (LM) und postulierte, dass asymmetrisch pigmentierte Haarfollikel, braun-schwarze rhomboidale Linien sowie graue Punkte zu den ersten Veränderungen der LM gehören. Eine rezente Studie [6] an über 1000 Läsionen untersuchte die Genauigkeit der bisher beschriebenen Kriterien von In-situ-Melanomen im Vergleich zu Nävi und nichtmelanozytären Tumoren. Die Autoren beschrieben, dass irreguläre hyperpigmentierte Areale, „prominent skin markings“ (lineäre, sich kreuzende Furchen, die heller sind als die Pigmentierung der Läsion), ein atypisches Netzwerk, ausgeprägte Regressionszeichen (blau-graue Punkte und/oder weiße Areale) und gewinkelte Linien die maßgebendsten Kriterien für In-situ-Melanome sind.

Zusammenfassend kann festgehalten werden, dass oben beschriebene dermatoskopische Muster sehr nützlich sind, um LPLK von frühen Melanomen zu unterscheiden (Tab. 1). Ein diagnostisches Dilemma stellen allerdings weiterhin vollständig regressive Läsionen dar, wobei festgehalten werden muss, dass regelmäßig verteilte graue Punkte gleicher Größe als einziges Muster eher für eine LPLK sprechen.

**Table 1:** 

Kriterium	Lichen-planus-artige Keratose	Lentigo maligna
Ein Muster (strukturlos, Punkte, gewinkelte Linien)	++	+
Mehrere Muster	+ (strukturlos + Punkte/strukturlos + gewinkelte Linien)	++
Muster einer seborrhoischen Keratose/Lentigo solaris	++	–
Schuppung	++	–
Orangefarbene Areale	++	–
Hellbraunes Pseudonetzwerk	+++	–
Punktgefäße	++	–
Asymmetrisch pigmentierte Haarfollikel	–	+++
Rhomboidale Linien	–	+++
Exzentrische hyperpigmentierte Areale	–	++
„Prominent skin markings“	–	++
Weiße/narbenartige Regression	–	+
Blau-graue Punkte	+++ (je nach Stadium; grobkörnig; typisch als alleiniges Kriterium)	++
Tastbefund	Erhaben/fettig	Flach

+ manchmal vorhanden, ++ sehr wahrscheinlich vorhanden, +++ nahezu immer vorhanden/diagnostisch, **–** nicht vorhanden
